# Estimating the burden of disease in chronic pain with and without neuropathic characteristics: Does the choice between the EQ-5D and SF-6D matter?

**DOI:** 10.1016/j.pain.2014.07.001

**Published:** 2014-10

**Authors:** Nicola Torrance, Kenny D. Lawson, Ebenezer Afolabi, Michael I. Bennett, Michael G. Serpell, Kate M. Dunn, Blair H. Smith

**Affiliations:** a Medical Research Institute, University of Dundee, Dundee, Scotland, UK; b Centre for Research Excellence in the Prevention of Chronic Conditions in Rural and Remote Populations, James Cook University, Townsville City, Australia; c Research Institute for Primary Care & Health Sciences, Keele University, Staffordshire, England, UK; d Academic Unit of Palliative Care, University of Leeds, Leeds, England, UK; e School of Medicine, University of Glasgow, Glasgow, Scotland, UK

**Keywords:** Chronic pain, EQ-5D, Health-related quality of life, Health utilities, Neuropathic pain, SF-6D, S-LANSS

## Abstract

The EQ-5D and Short Form (SF)12 are widely used generic health-related quality of life (HRQoL) questionnaires. They can be used to derive health utility index scores, on a scale where 0 is equivalent to death and 1 represents full health, with scores less than zero representing states “worse than death.” We compared EQ-5D or SF-6D health utility index scores in patients with no chronic pain, and chronic pain with and without neuropathic characteristics (NC), and to explore their discriminant ability for pain severity. Self-reported health and chronic pain status was collected as part of a UK general population survey (n = 4451). We found moderate agreement between individual dimensions of EQ-5D and SF-6D, with most highly correlated dimensions found for mental health and anxiety/depression, role limitations and usual activities, and pain and pain/discomfort. Overall 43% reported full health on the EQ-5D, compared with only 4.2% on the SF-6D. There were significant differences in mean utilities for chronic pain with NC (EQ-5D 0.47 vs SF-6D 0.62) and especially for severe pain (EQ-5D 0.33 vs SF-6D 0.58). On the EQ-5D, 17% of those with chronic pain with NC and 3% without NC scored “worse than death,” a state which is not possible using the SF-6D. Health utilities derived from EQ-5D and SF-12/36 can discriminate between group differences for chronic pain with and without NC and greater pain severity. However, the instruments generate widely differing HRQoL scores for the same patient groups. The choice between using the EQ-5D or SF-6D matters greatly when estimating the burden of disease.

## Summary

Chronic pain with neuropathic characteristics is associated with significantly lower EQ-5D and Short Form 6D health utilities scores, with 17% reporting health states “worse than death”.

## 1 Introduction

Chronic pain is common, affecting up to half of the adult population [17], [47]. Approximately 20% of the adult European population has significant chronic pain, and 7% to 8% of the population has chronic pain with neuropathic features [7], [10], [47]. Health-related quality of life (HRQoL) is significantly poorer in people with chronic pain than in those without [42], and poorer in people with neuropathic pain than in those with nonneuropathic pain [2], [16], [25], [43], [48].

HRQoL measures can be categorized into disease-specific and generic measures. It is important that these measures are valid, appropriate to the disease, and particularly for clinical trials, sensitive to detect changes. Although a disease-specific measure for neuropathic pain has been developed [39], many studies use a generic HRQoL measure alongside clinical assessment or a validated neuropathic pain screening tool [2], [43]. A single summary score of overall HRQoL is generated by weighting responses to mental and physical health states by their perceived importance, using patient or general population preferences. This single summary score can be used to compare the impact of different conditions and how they vary across populations and to quantify the effectiveness of interventions. Economists term such scores health utilities or health utility index scores. Health utilities are measured on a scale where 0 represents a health state equivalent to death and 1 represents full health, with the potential for values less than zero representing states “worse than death” (WTD). A concern in only using generic measures is whether they are sensitive enough to discriminate between patients in whom important but complex differences might be detected by more specific measures.

The EQ-5D [18] and Short Form (SF) 12/36 [53], [54] questionnaires are widely used generic HRQoL measures. They are a common means of generating health state values using an algorithm to derive health utility scores (SF-6D from SF-12/36) [5] and are used by economists to calculate quality adjusted life years (QALYs) [35], [49], [57] and in economic evaluations to evaluate the cost effectiveness of health care interventions.

The association between neuropathic pain conditions and health utilities has been described in a multicenter European cross-sectional survey [33] and a systematic review [16], both finding a significant relationship between increasing pain severity and reduced HRQoL. The EQ-5D has been used to measure HRQoL of patients with specific neuropathic pain diagnoses [16], but there are few published studies that have used SF-6D in patients with neuropathic pain [29], despite its widespread use and acceptance. Studies have directly compared EQ-5D and SF-6D utilities in patients with chronic painful conditions, such as arthritis [8], [14], [23], [26], [31], [37], [57], low back pain [8], [14], [37], and nonspecific neck pain [55], with most finding moderate levels of agreement. These patients were generally recruited according to strict criteria and are unlikely to represent HRQoL associated with these conditions in general populations and primary care, where most chronic and neuropathic pain is treated and managed [46].

This study collected data on HRQoL and chronic pain status as part of a large UK general population questionnaire survey [46]. We compared EQ-5D and SF-6D health utility index scores in individuals with chronic pain, with and without neuropathic characteristics. We also explored the ability of these generic measures to discriminate between the health utilities of patients with different pain severities.

## 2 Methods

### 2.1 Sample selection

In the UK, around 96% of the population is registered with a general practitioner (family doctor, GP) [36]; a GP practice population therefore approximates to a general population sample. This study surveyed 10,000 individuals in 5 UK locations, with 2 GP practices in each locality generating a random sample of 1000 registered adult patients. Each practice’s electronic register was used to generate a random sample of patients over age 18 years. The sample list was then screened by the GPs in each practice, to exclude patients in whom inquiry might be insensitive or inappropriate (for example, in terminal illness or with severe learning difficulties). Details of the sample selection procedures have been reported previously [46].

### 2.2 Patient questionnaire

Individuals in the study sample were mailed a self-complete questionnaire that contained demographic items (age, sex, smoking, marital and employment status, educational attainment, and home ownership as a proxy for social class [44]), HRQoL measures including the SF-12 and EQ-5D questionnaires, chronic pain identification and severity questions, and the Self-Complete Leeds Assessment of Neuropathic Symptoms and Signs (S-LANSS) questionnaire, which is used to identify pain with neuropathic characteristics (NC) [4].

#### 2.2.1 Pain ascertainment and characteristics

Chronic pain was identified by affirmative answers to 2 questions: (1) Are you currently troubled by pain or discomfort, either all the time or on and off? (2) Have you had this pain or discomfort for more than 3 months? [24] Identical case identification questions have been used in previous population-based research on chronic pain [13], [17], [47].

The S-LANSS questionnaire is a validated 7-item questionnaire including 5 questions about pain characteristics and 2 self-examination items; its responses are weighted to provide a maximum score of 24, with a score ⩾12 indicating pain with NC [4]. Pain severity was measured using the average pain intensity numeric rating scale (NRS) of the Chronic Pain Grade (CPG). This is a 0 to 10 NRS anchored at 0 for no pain and 10 for pain as bad as can be in the past 3 months [51].

#### 2.2.2 HRQoL and generating health utility scores

All respondents were asked to complete the SF-12 and EQ-5D HRQoL questionnaires before completion of the chronic pain screening questions. The SF-12 is a validated 12-item self-administered tool for measuring health status derived from the SF-36 [53]. The SF-12 has been used in large general population questionnaire studies of chronic pain [2], [12], [29], [30] and in studies of specific neuropathic pain conditions, such as postherpetic neuralgia [6]. SF-12 scores can be calculated in 8 health domains: physical functioning, role physical, bodily pain, general health, vitality, social functioning, role emotional, and mental health. To generate a single health utility score from the SF-12, we used the standard SF-6D algorithm [9]. This algorithm involves preference weighting of 6 of the SF-12 question responses (3 physical health and 3 mental health) by the desirability for different health states. These preference weights were derived from a survey representative of the UK general population [9]. Summing across weighted question responses generated a health utility score for each respondent [9]. The SF-6D generates a score on a 0.29 to 1.00 scale, with 1.00 indicating full health. The SF-6D can define 18,000 and 7500 health states for the SF-6D (SF-36) and SF-6D (SF-12), respectively [56].

The EQ-5D is a generic measure of health status and defines health in terms of 5 dimensions: mobility, self-care, usual activities (work, study, housework, family, or leisure), pain or discomfort, and anxiety or depression, and is well validated in population studies [18], [27]. A preference-based set of weights (or algorithm) is used to calculate a single EQ-5D index-based utility score. The EQ-5D generates a score on a −0.59 to 1.00 scale, with 1.00 indicating full health and 0 equal to death. The negative EQ-5D utility values theoretically correspond to health states valued as WTD. (Negative utility values are not available with SF-6D.) The EQ-5D can define 243 distinct states [56].

### 2.3 Data analysis

A complete case analysis was conducted. Descriptive statistics show the sociodemographic characteristics of the whole study sample and by defined pain group (no chronic pain; chronic pain without neuropathic characteristics (S-LANSS < 12; chronic pain without NC); chronic pain with neuropathic characteristics (S-LANSS ⩾ 12; chronic pain with NC). Linear regression analysis was conducted to determine differences in health utilities among the 3 defined pain subpopulations (no chronic pain vs chronic pain without NC, no chronic pain vs chronic pain with NC, chronic pain without NC vs chronic pain with NC) with adjustment for all significant demographic variables.

The data analysis was divided into 3 elements: an assessment of the correlation between EQ-5D and SF-6D dimensions, the range of observed health utility index scores, and the sensitivity of both instruments to detect differences between chronic pain types and degrees of pain severity.

#### 2.3.1 Correlation between EQ-5D and SF-6D dimensions

EQ-5D dimensions are what the respondents reported, and the EQ-5D health utility indices are derived using the algorithm. Similarly, SF-6D dimensions were obtained from the SF-12 responses, and health utility indices were calculated using the SF-6D algorithm. The first section of the analysis is based on EQ-5D and SF-6D self-reported dimensions with no reference to single index scores. This assessment of the degree of agreement between dimensions of the 2 instruments used Spearman rank correlations across the whole sample and by defined pain group. The value of the correlation coefficient can be interpreted thus: 1 is perfect, 0.7 to 0.9 is strong, 0.4 to 0.69 is moderate, 0.1 to 0.39 is weak, and 0 is no correlation [15]. We hypothesized that there would be correlations between the dimensions purporting to capture similar aspects in SF-12 and EQ-5D. These similar dimensions are physical functioning and mobility, physical functioning and usual activities, role limitations and usual activities, role limitations and anxiety/depression, social functioning and usual activities, pain and pain/discomfort, mental health and anxiety/depression, vitality and usual activities [55].

#### 2.3.2 Range of the health utility index scores

Further analysis then explored the health utility indices with basic descriptive statistics, including means, medians, and ranges. The level of agreement between EQ-5D and SF-6D index scores also was examined by calculating the intraclass correlation coefficient (ICC), using a 2-way mixed model based on absolute agreement, where the 2 measures are treated as a source of variability [8], [55]. The values of the ICC can theoretically range from 0 to 1, with a higher value indicating that less variance is due to other factors such as differences between observations. For the 2 instruments, the level used to interpret the ICC was 0.00 to 0.10 = virtually no agreement, 0.11 to 0.40 = slight, 0.41 to 0.60 = fair, 0.61 to 0.80 = moderate, and 0.81 to 1.00 = substantial agreement [41], [56]. Floor and ceiling effects (proportion of respondents with the best and worst possible theoretical scores, respectively) were also explored for both the EQ-5D and SF-6D indices.

#### 2.3.3 Discriminating health utility index scores among degrees of pain severity

In comparing the EQ-5D and SF-6D, a key criterion is whether the instruments are sensitive enough to discriminate differences in reported patients’ pain severity considered to be clinically meaningful. Because this was a cross-sectional study, we were unable to measure changes in utility scores over time. However, within an exploratory analysis, we were able to estimate the difference in utility scores for respondents with different levels of pain severity. This provides an indication of the potential change in utility scores that each instrument may detect if there was an intervention that moved patients between pain severity groups. This analysis is exploratory and intended to inform whether further research using a longitudinal study is necessary.

Patients were first divided into 3 categories of reported pain severity. We used clinically validated cut-points for pain severity to create categories of mild, 0 to 3; moderate, 4 to 6; and severe, 7 to 10 chronic pain on the average pain intensity NRS of the Chronic Pain Grade [51], [59]. We then compared mean utility scores from the EQ-5D and SF-6D across these 3 pain intensity groups as a means to infer discriminant ability regarding HRQoL. We also tested whether the difference in utility scores among pain groups is also meaningful to patients in terms of their perception of HRQoL. This was done by comparing the differences in utility scores to the minimally important difference (MID) according to Walters and Brazier [52], who in comparing the results of 11 longitudinal studies (the majority of which were chronic pain–related conditions, including back pain and arthritis) found the mean MID for EQ-5D to be 0.074 (range 0.011 to 0.140) and for SF-6D to be 0.041 (range 0.011 to 0.097) [52].

Because previous research has found HRQoL in individuals with neuropathic pain to be worse than in those with nonneuropathic pain of the same severity [43], we hypothesized that those respondents in the chronic pain with NC group would be more likely (than chronic pain without NC) to report WTD scores in EQ-5D and therefore to demonstrate a floor effect equivalent to worst possible health (⩽ 0) [55].

### 2.4 Ethics approval

The study was approved by North of Scotland Research Ethics Committee, REC reference number 09/S0802/103.

## 3 Results

### 3.1 Characteristics of study sample

In total, 10,000 postal questionnaires were mailed, with 347 returned as undelivered or unable to be completed due to illness or learning disability. Of the rest, 4541 completed questionnaires were returned, giving an overall corrected response rate of 47%. Further information on the respondents and the study sample has been reported previously [46].

Of the 4451 returned questionnaires, 4408 individuals completed both of the 2 screening questions for chronic pain status. Any chronic pain was reported by 2202 (48.5%; 95% confidence interval 47.0% to 49.9%) and 2206 respondents reported no chronic pain. S-LANSS questionnaires were incomplete in 192 of those with any chronic pain, and these individuals were excluded from further categorization into the chronic pain with or without neuropathic pain groups for analysis. Therefore 1611 individuals were categorized as chronic pain without NC and 399 as chronic pain with NC (S-LANSS ⩾ 12).

Completion rates were high for both HRQoL questionnaires, with the EQ-5D completed by 4349 (95.8%) and the SF-12 completed by 4176 (92.0%) of all respondents. The characteristics of the whole study sample and by pain group are shown in Table 1. There were significant differences in all of the measured sociodemographic characteristics between respondents reporting chronic pain with NC and those reporting chronic pain without NC except for age (mean (SD) 56.0 (15.4) years vs 56.3 (15.3) years, *P* = .673). Individuals with chronic pain with NC were more likely to be women, no longer married, and living in council rented accommodation than individuals whose chronic pain that did not have NC. They were also more likely to be unable to work due to illness or disability, to have no educational qualifications, and to be smokers. Pairwise comparisons between pain groups found those reporting chronic pain with NC to have significantly lower EQ-5D and SF-6D mean utility scores than the no Chronic pain and chronic pain without NC groups (*P* < .001). Linear regression found the significant differences in health utilities between the pain groups persisted (*P* < .001) after adjustment for all significant sociodemographic variables (sex, marital status, employment, housing, general health, education, and smoking).

**Table 1:** Sample characteristics of respondents by chronic pain group, n (%).

	Whole sample (n = 4541)	No chronic pain (n = 2206)	Chronic pain without NC (n = 1611)	Chronic pain with NC (n = 399)
*Age, n (%)*				
18 to 39 y	968 (21.5)	654 (28.5)	234 (14.7)	60 (15.3)
40 to 59 y	1789 (39.4)	928 (40.4)	622 (39.0)	167 (42.5)
60+ y	1738 (38.3)	692 (30.1)	740 (46.4)	166 (42.2)

*Gender*				
Men	1928 (42.5)	1016 (44.3)	684 (42.5)	146 (36.8)
Women	2609 (57.5	1280 (55.7)	925 (57.5)	251 (63.2)

*Marital status*				
Never married	634 (14.0)	382 (16.6)	166 (10.4)	55 (14.0)
Living as married	3190 (70.6)	1614 (70.3)	1194 (74.5)	245 (62.2)
No longer married	693 (15.3)	290 (12.6)	243 (15.2)	94 (23.9)

*Housing tenure*				
Owned/mortgaged	3657 (81.2)	1914 (85.4)	1321 (82.6)	261 (66.4)
Council rent	527 (11.7)	190 (8.5)	188 (11.8)	92 (23.4)
Private rent/other	322 (7.1)	178 (7.8)	91 (5.7)	40 (10.2)

*Employment*				
Employed	2486 (55.1)	1483 (64.6)	774 (48.3)	151 (38.4)
Retired	1456 (32.3)	562 (24.5)	638 (39.9)	137 (34.9)
Unable to work	199 (4.4)	28 (1.2)	70 (4.4)	77 (19.6)
Not employed/other	369 (8.2)	212 (9.2)	119 (7.4)	28 (7.1)

*Education*				
No qualifications	862 (19.3)	323 (14.1)	345 (21.7)	115 (29.6)
Secondary school/equivalent	1785 (40.1)	933 (40.6)	599 (37.7)	165 (42.5)
Higher education	1808 (40.6)	1010 (44.0)	644 (40.6)	108 (27.8)

*Smoking*				
Smoker	798 (17.6)	372 (16.2)	264 (16.4)	105 (26.4)
Ex-smoker	1396 (30.9)	632 (27.5)	580 (36.1)	109 (27.5)
Never smoked	2329 (51.5)	1288 (56.1)	761 (47.3)	183 (46.1)

*Perceived health status*				
Excellent	571 (12.7)	475 (20.8)	88 (5.5)	7 (1.8)
Very good	1611 (35.7)	1022 (44.7)	470 (29.4)	65 (16.6)
Good	1436 (31.8)	633 (27.7)	626 (39.1)	122 (31.1)
Fair	649 (14.4)	149 (6.5)	316 (19.8)	105 (26.8)
Poor	246 (5.5)	9 (0.4)	100 (6.2)	93 (23.7)

SF-6D, mean (SD)	0.767 (0.15)	0.826 (0.12)	0.728 (0.14)	0.619 (0.15)
EQ-5D index score, mean (SD)	0.794 (0.27)	0.932 (0.13)	0.702 (0.25)	0.468 (0.36)
EQ-VAS, mean (SD)	77.82 (19.38)	85.31 (13.5)	73.35 (19.3)	59.67 (24.0)

Pairwise comparisons (independent samples *t* tests) for SF-6D and EQ-5D found *P* < .001 for no chronic pain vs chronic pain without NC, no chronic pain vs chronic pain with NC, chronic pain without NC vs chronic pain with NC.

NC = neuropathic characteristics.

### 3.2 Correlation between EQ-5D and SF-6D dimensions

Table 2 presents the relationships between EQ-5D and the SF-6D dimensions as measured by Spearman correlation coefficients for the whole dataset, and then separately for the 2 chronic pain groups. The similar dimensions that were expected to have the highest correlations are underlined [8], [55] with the 5 actual most correlated dimensions shown in boldface type. We identified 8 paired dimensions that were expected to be among the highest correlations. Of these, the highest correlations were: between mobility and usual activities (EQ-5D) with physical functioning (SF-6D), between usual activities (EQ-5D) and role limitations and pain (SF-6D), between pain/discomfort (EQ-5D) and pain (SF-6D), and between anxiety/depression (EQ-5D) mental health (SF-6D). These all had a correlation coefficient >0.60, with the strongest correlation (0.75) found between the 2 pain dimensions in the whole sample. These results provide evidence for agreement, but lower correlations were found between other similar dimensions, notably between anxiety/depression (EQ-5D) and mental health (SF-6D). The 2-way mixed ICC for the whole sample was 0.61, which suggests moderate agreement between the 2 measures, with the ICC 0.44 for the chronic pain with NC and 0.57 for chronic pain without NC groups.

**Table 2:** The correlation between EQ-5D and SF-6D dimensions (Spearman rank correlation).

SF-6D	EQ-5D				
	Mobility	Self care	Usual activities	Pain/discomfort	Anxiety/depression
*Whole sample, n = 4541*					
Physical functioning	**0.65**	0.44	**0.64**	0.55	0.30
Role limitations	0.63	0.45	**0.71**	0.60	0.36
Social functioning	0.46	0.41	0.56	0.44	0.56
Pain	0.61	0.41	**0.65**	0.75	**0.35**
Mental health	0.24	0.26	0.33	0.29	**0.64**
Vitality	0.44	0.35	0.50	0.42	0.42

*Chronic pain without NC, n = 1611*					
Physical functioning	**0.64**	0.40	**0.65**	0.46	0.21
Role limitations	0.59	0.42	**0.69**	0.46	0.26
Social functioning	0.39	0.38	0.50	0.34	0.27
Pain	0.56	0.39	**0.61**	0.55	0.27
Mental health	0.15	0.22	0.26	0.19	**0.66**
Vitality	0.36	0.33	0.46	0.31	0.40

*Chronic pain with NC, n = 399*					
Physical functioning	**0.67**	0.54	**0.60**	0.50	0.32
Role limitations	0.60	0.57	**0.71**	0.53	0.41
Social functioning	0.44	0.51	0.51	0.43	0.57
Pain	0.53	0.51	0.57	**0.66**	0.39
Mental health	0.25	0.39	0.37	0.30	**0.73**
Vitality	0.41	0.43	0.50	0.36	0.43

The 5 most correlated dimensions are indicated in boldface type.

The underlined correlations were identified as purporting to capture similar aspects of quality of life.

NC = neuropathic characteristics.

Further analysis was conducted into the responses to the pain questions in both EQ-5D and SF-6D (SF-12), where 94.3% (366 of 388) and 71.2% (282 of 396) of those with chronic pain with NC reported moderate to extreme pain, respectively, compared with 80.7% (1278 of 1584) and 39.2% (629 of 1605) of those with chronic pain without NC.

### 3.3 Range of the health utility index scores

The mean EQ-5D and the SF-6D scores are shown in Table 3. For the whole sample, the EQ-5D mean score was greater than the SF-6D by 0.02; however, for the 2 pain groups the mean SF-6D scores were greater, by 0.03 in the chronic pain without NC group, and by 0.15 in the chronic pain with NC. Overall, median scores were higher than mean, indicating a skewed distribution for both indices, except for the SF-6D index for the chronic pain with NC group (mean 0.62, median 0.60). Floor effects were small in both of the measures, and we did not reach the absolute theoretical floor effect of EQ-5D (−0.594), with the lowest score found to be −0.371.

**Table 3:** Distribution of EQ-5D and SF-6D indices.

	No. of items	Theoretical range	Observed range	Floor effect,⁎ n (%)	Ceiling effect,† n (%)	Mean (SD)	Median (IQR)	ICC of indices
*Whole sample, n = 4541*								
EQ-5D, n = 4349	5	−0.594 to 1	−0.371 to 1.00	119 (2.7)	1850 (42.5)	0.79 (0.27)	0.85 (0.73 to 1.00)	0.61
SF-6D, n = 4176	6	0.29 to 1	0.345 to 1.00	9 (0.2)	174 (4.2)	0.77 (0.15)	0.80 (0.66 to 0.92)	

*Chronic pain without NC, n = 1611*								
EQ-5D, n = 1551	5	−0.594 to 1	−0.235 to 1.00	54 (3.8)	239 (15.4)	0.70 (0.25)	0.76 (0.69 to 0.80)	0.57
SF-6D, n = 1499	6	0.29 to 1	0.345 to 1.00	1 (0.1)	18 (1.2)	0.73 (0.14)	0.72 (0.62 to 0.86)	

*Chronic pain with NC, n = 399*								
EQ-5D, n = 373	5	−0.594 to 1	−0.371 to 1.00	64 (17.2)	14 (3.8)	0.47 (0.36)	0.62 (0.89 to 0.73)	0.44
SF-6D, n = 358	6	0.29 to 1	0.345 to 1.00	5 (1.4)	3 (0.8)	0.62 (0.15)	0.60 (0.52 to 0.71)	

IQR = interquartile range; ICC = intraclass correlation coefficient, NC = neuropathic characteristics.

⁎ Floor effect, less than 0 (worse than death) for EQ-5D and minimum value for SF-6D = 0.345.

† Ceiling effect = 1 for both instruments.

We observed 119 respondents with a score <0 or WTD on the EQ-5D (2.7% of the whole sample). Only 1 of these individuals reported no chronic pain, 54 individuals reported chronic pain without NC (from total n = 1551), representing 45.4% of all WTD scores and 3.4% of chronic pain without NC. The remaining 64 individuals with WTD utility scores reported chronic pain with NC, representing 53.8% of all WTD scores and 17.2% of the chronic pain with NC group (n = 64 of 373 with complete data). The majority of respondents who were WTD in both pain groups reported severe average pain (⩾7 of 10), 98.1% (n = 53 of 54) of the chronic pain without NC and 83.6% (n = 51 of 1) of chronic pain with NC (χ^2^ test, *P* = .03). The lowest score observed for SF-6D was 0.345 in 1.4% (n = 5 of 358) of those with chronic pain with NC, none of the respondents reached the theoretical lowest threshold of 0.29.

**Fig. 1:**
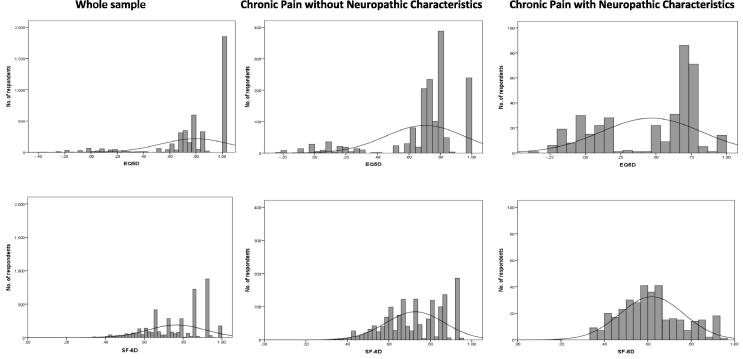
Frequency distributions by pain group.

The EQ-5D scores were negatively skewed with a ceiling effect that was most apparent in the whole general population sample, where 42.5% (n = 1850) attained a maximum score of 1 (Table 3). The highest possible score on EQ-5D was also found in 15.4% of those with chronic pain without NC and in 3.8% of the chronic pain with NC group, and there were few scores between 0.88 and 1. This compares to a ceiling effect in the SF-6D found in 4.2%, 1.2%, and 0.8% in the whole sample, and the chronic pain without and with NC groups, respectively. The SF-6D is also negatively skewed in the whole sample, but then these scores approximate a normal distribution in the 2 pain groups because the SF-6D was more positively skewed and is limited at the floor (0.29). The distribution of EQ-5D scores was distributed across the range of scores in the chronic pain groups. It also appears that the SF-6D was more continuous, whereas the EQ-5D appeared more discrete with gaps between states, with an apparent bimodal distribution in both the chronic pain groups. The frequency distributions of the utility scores for the whole sample and the 2 pain groups are shown in Fig. 1.

### 3.4 Discriminating health utility scores by level of pain severity

Table 4 shows the mean EQ-5D and SF-6D utilities by mild, moderate, and severe average pain. Of the total 2010 respondents with chronic pain, 1972 completed the average pain NRS, 22.6% (n = 445) reporting mild chronic pain, 41.7% (n = 822) reporting moderate chronic pain, and 35.8% (n = 705) reporting severe chronic pain. Overall, those with severe pain had the lowest utilities for both EQ-5D and SF-6D as expected; furthermore, the EQ-5D utilities were lower in chronic pain (with and without NC) compared with the SF-6D. In the chronic pain with NC group, mean EQ-5D health utilities for those reporting mild pain intensity was 0.72, for those with moderate pain the mean was 0.63, and for those with severe pain it was 0.33. Therefore, the mean between-group differences (for pain intensity) were 0.09 (moderate-mild) and 0.39 (severe-moderate), and above the published mean MID of 0.074 [52]. For SF-6D utilities, in the chronic pain with NC group, the mean difference in health utilities between those with moderate and mild pain was 0.08, with the same mean difference between severe and moderate pain intensity, and also above the mean MID of 0.041 [52]. Notably, in comparing the EQ-5D and SF-6D, utility scores as measured by the EQ-5D were considerably lower in those with any severe pain (mean 0.48 vs 0.63), and the EQ-5D showed approximately twice the range of between-group differences (0.55 vs 0.33) compared with the SF-6D scores (0.65 vs 0.58).

**Table 4:** Health utilities by pain severity, mean (SD).

	Mild (n = 445)	Moderate (n = 822)	Severe (n = 705)	Mean difference mild-moderate	Mean difference moderate-severe	Mean difference mild-severe	*P* value⁎
*Any chronic pain*							
EQ-5D	0.82 (0.17)	0.72 (0.20)	0.48 (0.35)	0.10	0.24	0.34	<.001
SF-6D	0.79 (0.12)	0.73 (0.13)	0.63 (0.15)	0.06	0.10	0.16	<.001

*Chronic pain without NC*							
EQ-5D	0.83 (0.16)	0.74 (0.17)	0.55 (0.32)	0.09	0.19	0.28	<.001
SF-6D	0.79 (0.11)	0.74 (0.13)	0.65 (0.15)	0.05	0.09	0.14	<.001

*Chronic pain with NC*							
EQ-5D	0.72 (0.24)	0.63 (0.27)	0.33 (0.36)	0.09	0.30	0.39	<.001
SF-6D	0.74 (0.13)	0.66 (0.14)	0.58 (0.14)	0.08	0.08	0.16	<.001

Mean Minimally Important Difference (MID) for EQ-5D 0.074 and mean MID for SF-6D 0.041 (Walters & Brazier, 2005 [52]).

NC = neuropathic characteristics.

⁎ ANOVA for mild, moderate and severe pain by pain group.

## 4 Discussion

This study compared the health utility scores derived from 2 widely used generic HRQoL measures, the EQ-5D and SF-6D, in a large general UK population sample focusing on chronic pain with and without neuropathic characteristics. We looked at the agreement between instruments in measuring individual health dimensions, the scoring range of the health utility scores, and whether scores could detect at least an MID between clinically meaningful differences in pain. Both instruments were able to discriminate important differences in pain groups and in pain intensity. Among those with chronic neuropathic pain, 17% had HRQoL scores equivalent to WTD on the EQ-5D.

### 4.1 Key findings and implications

#### 4.1.1 Comparison between individual dimensions

Overall, we found only moderate agreement between individual dimensions. The most highly correlated dimensions were mental health and anxiety/depression and role limitations and usual activities. The pain and pain/discomfort dimensions were more highly correlated in the chronic pain with NC group (compared to without NC).

#### 4.1.2 Health utility scores: range, floor, and ceiling effects

We confirmed the considerable ceiling effect of EQ-5D observed in previous general population surveys [5], [14], [38], with 43% of our whole sample reporting full health, compared with only 4.2% who were classified in full health on the SF-6D. The EQ-5D appears insensitive at the top (healthy end) of the scale, and a gap exists between 0.88 and 1. The SF-6D does not seem to have a ceiling effect and may capture smaller health changes toward the top of the scale [11]. However, this may be less relevant in chronic neuropathic pain, in which the proportion of respondents attaining a maximum utility score with EQ-5D was relatively small (only 3.8% reported full health with EQ-5D and 0.8% with SF-6D).

In total, 17% of chronic pain with NC and 3% of chronic pain without NC respondents had a score of below 0 or WTD on EQ-5D. Almost all of those with a WTD score also reported severe pain (⩾7 of 10). Other studies of rheumatoid arthritis (RA) report similar findings: in a study of patients with established RA [1], 17% had WTD scores at baseline (before biological therapy) and 7% at 12-month follow-up. Extreme pain scores were strongly associated with a state WTD in a study of early arthritis, in which 11% had a negative EQ-5D score [21], and in patients with RA [22], 9% of trial participants had states WTD. In these studies, extreme pain/discomfort was the key EQ-5D domain associated with a WTD state, plus moderate problems in ⩾3 other domains [22]. Whitehurst et al. (2011) [56] suggest that the EQ-5D may be better suited to capture the magnitude of severity for poorer health states. Notably, however, the 20% of patients who on the EQ-5D had a score ⩽0 did not actually reach the SF-6D floor of 0.29. This raises interesting issues regarding the true HRQoL state of such patients. For instance, if such states are really considered as WTD, as estimated by EQ-5D, it may be legitimately expected that the utility scores of SF-6D in these patients would have clustered at the SF-6D floor of 0.29. However, the mean score of patients with chronic pain with neuropathic characteristics was 0.34.

#### 4.1.3 Mean scores between patient groups

There were differences in the mean utility scores for the 2 instruments, with EQ-5D utility scores higher in the whole sample and the no chronic pain groups, whereas SF-6D scores were higher in both chronic pain groups. This is most striking in chronic pain with NC, in which the mean SF-6D scores were 0.15 higher. Other studies have found the average differences in means to be around 0.05 [8], [38] although this varies, with higher mean differences (0.15) reported in studies of severe pain conditions such as severe knee osteoarthritis [37], [57] and inflammatory RA [23]. These results suggest that it may be unreliable, perhaps even invalid, to compare studies of severe pain-related conditions that have used different health utility measures.

#### 4.1.4 Inferring the potential sensitivity to detect a change in utility

In an exploratory exercise, we attempted to use these cross-sectional data to infer the potential sensitivity of the 2 instruments if there was an intervention that led to a clinically meaningful reduction in pain. Respondents were classified according to clinically validated cut-points for mild, moderate, and severe chronic pain [59]. It has been suggested that a clinically important outcome would be to reduce a patient’s level of pain down to no worse than mild [34]. We estimated the difference in mean utility scores between different pain severity groups and compared this to the MID [52]. We found the mean between-group differences were above the MID for both scores. However, the differences were higher using the EQ-5D. In particular, the difference in utility between moderate-severe pain was 5-fold, 0.39 using EQ-5D and 0.08 using SF-6D. If such findings were to be found in a randomized trial, in which a patient’s pain severity reduced from severe to moderate, this would have substantial implications regarding the cost effectiveness (cost-utility analysis) of the intervention. This analysis points to the need for further research using trial and longitudinal data. This is discussed further in Section 4.4. Other studies that compared the utility estimates of EQ-5D and SF-6D in randomized trials found the choice between the instruments to be very important regarding the cost-utility estimates produced [8], [26], [40].

### 4.2 Study limitations

This study comprises a large dataset derived from a random sample of adults generated from GP practices in the UK. The relatively low response rate is similar to previous surveys of pain prevalence [10], [32] and is an increasingly common problem in epidemiological research [20], [28], [32], [50]. However, the prevalence of chronic pain and chronic pain with NC was similar to that in other population studies with higher response rates [47], [58]. In relation to possible response bias and practicality, completion rates were high for both the EQ-5D and SF-12, with slightly higher response rate in favor of the EQ-5D. Similar differences in completion rates have been reported elsewhere [3], [55].

### 4.3 Future research directions

Researchers and clinicians should consider using generic health utility instruments in pain-related burden of disease studies. The rationale for generating health utility scores is that it provides a generic, preference-weighted index that enables the severity of different conditions to be estimated consistently. The ultimate intention is to assist health care planners to allocate resources on a consistent and transparent basis among different diseases and interventions.

There are a number of utility instruments [9], [18], [19] commonly used in clinical practice and research. The findings from this study, that the choice between EQ-5D and SF-6D results in major differences in the estimation of utility scores for severe chronic (and neuropathic) pain, questions the validity of comparing studies that have used different instruments. This discordance warrants further investigation in other pain populations.

Our exploratory analysis, using cross-sectional data, compared the mean utility scores between the instruments for patients classified by different cut-points for pain severity. The difference between the mean utility scores of patients with severe and moderate pain was 5 times greater when estimated using EQ-5D compared with SF-6D. Therefore, the choice between using the EQ-5D and SF-6D not only may be important in estimating the absolute burden of disease, but also may have major implications regarding the economic evaluation of interventions that take a cost-utility approach. It is important to compare these instruments in longitudinal studies to assess their sensitivity to detect changes in pain severity.

Finally, it is important to note that cost utility analysis (cost effectiveness analysis) uses measures of relative change, in which improvement in health utilities scores are valued equally, irrespective of the level of utilities postintervention [35]. This potentially raises concerns if an intervention involving patients with WTD scores (as measured by EQ-5D) improves health utilities but the patients remain in a state WTD. The National Institute for Health and Clinical Excellence recommends the use of general population preferences when generating utility index scores [35], meaning that it is not actually the patients themselves who would prefer death but the general population who score these health states. As people with chronic pain come to accept and adjust over time [45], the meaning and interpretation of WTD seems to raise both ethical and practical considerations regarding measuring burden of disease and in the assessment of utility.

Overall, the rationale for attempting to generate utility index scores to generate a consistent HRQoL outcome measure is important. However, there are a number of practical issues that this study has raised, in common with other research [8], [26], [40].

### 4.4 Conclusions

The measurement of HRQOL is important in chronic pain research, and health utilities derived from generic instruments such as EQ-5D and SF-12/36 can discriminate among group differences for chronic pain with and without NC and greater pain severity. This study demonstrates the substantial lack of agreement between EQ-5D and SF-6D when estimating the burden of disease for severe chronic pain. Future research should include longitudinal and clinical studies to test the validity of utility scores to understand the true health state of patients and also to assess the sensitivity of scores to detect changes in HRQoL as individuals’ pain severity ratings change. The choice between the instruments has substantial implications regarding the estimation of HRQoL in chronic pain patients.

## Conflict of interest statement

N.T., K.L., E.A., and K.D. have no conflicts of interest. B.S. has received occasional lecture and consultancy fees, on behalf of his institution, from companies involved in the manufacture of drugs used in treating neuropathic pain. M.S has received research support, consulting fees, or honoraria in the past 3 years from Astellas, Astra Zenica, Grünenthal, GW Pharmaceuticals, Lilly, NAPP, and Pfizer. M.B. has received consultancy fees and lecturer honoraria from Pfizer, Astellas, and Grunenthal in the last 3 years. Kate Dunn is supported by the 10.13039/100004440Wellcome Trust [083572]. The authors assert no personal pecuniary or other conflict of interest in the writing of this article. No writing assistance was used in the production of this article.
